# Anti-Inflammatory Activities of Pentaherbs formula and Its Influence on Gut Microbiota in Allergic Asthma

**DOI:** 10.3390/molecules23112776

**Published:** 2018-10-26

**Authors:** Miranda Sin-Man Tsang, Sau-Wan Cheng, Jing Zhu, Karam Atli, Ben Chung-Lap Chan, Dehua Liu, Helen Yau-Tsz Chan, Xiaoyu Sun, Ida Miu-Ting Chu, Kam-Lun Hon, Christopher Wai-Kei Lam, Pang-Chui Shaw, Ping-Chung Leung, Chun-Kwok Wong

**Affiliations:** 1 Institute of Chinese Medicine and State Key Laboratory of Research on Bioactivities and Clinical Applications of Medicinal Plants, The Chinese University of Hong Kong, Hong Kong, China; tsangsinman0128@gmail.com (M.S.-M.T.); sw_cheng@cuhk.edu.hk (S.-W.C.); benchan2011a@gmail.com (B.C.-L.C.); dehua.cau@gmail.com (D.L.); pcshaw@cuhk.edu.hk (P.-C.S.); pingcleung@cuhk.edu.hk (P.-C.L.); 2 Department of Chemical Pathology, The Chinese University of Hong Kong, Prince of Wales Hospital, Hong Kong, China; vivic5015@163.com (J.Z.); karamatli25@gmail.com (K.A.); chanyautsz@hotmail.com (H.Y.-T.C.); XiaoyuSUN@link.cuhk.edu.hk (X.S.); idachu1219@gmail.com (I.M.-T.C.); 3 Department of Paediatrics, The Chinese University of Hong Kong, Prince of Wales Hospital, Hong Kong, China; ehon@cuhk.edu.hk; 4 State Key Laboratory of Quality Research in Chinese Medicines, Macau Institute for Applied Research in Medicine and Health, Macau University of Science and Technology, Macau, China; wklam@must.edu.mo; 5 School of Life Sciences, The Chinese University of Hong Kong, Hong Kong, China; 6 Li Dak Sum Yip Yio Chin R & D Centre for Chinese Medicine, The Chinese University of Hong Kong, Hong Kong, China

**Keywords:** allergic asthma, cytokines, gut microbiota, regulatory T cells, short-chain fatty acids

## Abstract

Allergic asthma is a highly prevalent airway inflammatory disease, which involves the interaction between the immune system, environmental and genetic factors. Co-relation between allergic asthma and gut microbiota upon the change of diet have been widely reported, implicating that oral intake of alternative medicines possess a potential in the management of allergic asthma. Previous clinical, in vivo, and in vitro studies have shown that the Pentaherbs formula (PHF) comprising five traditional Chinese herbal medicines Lonicerae Flos, Menthae Herba, Phellodendri Cortex, Moutan Cortex, and Atractylodis Rhizoma possesses an anti-allergic and anti-inflammatory potential through suppressing various immune effector cells. In the present study, to further investigate the anti-inflammatory activities of PHF in allergic asthma, intragastrical administration of PHF was found to reduce airway hyperresponsiveness, airway wall remodeling and goblet cells hyperplasia in an ovalbumin (OVA)-induced allergic asthma mice model. PHF also significantly suppressed pulmonary eosinophilia and asthma-related cytokines IL-4 and IL-33 in bronchoalveolar lavage (BAL) fluid. In addition, PHF modulated the splenic regulatory T cells population, up-regulated regulatory interleukin (IL)-10 in serum, altered the microbial community structure and the short chain fatty acids content in the gut of the asthmatic mice. This study sheds light on the anti-inflammatory activities of PHF on allergic asthma. It also provides novel in vivo evidence that herbal medicines can ameliorate symptoms of allergic diseases may potentially prevent the development of subsequent atopic disorder such as allergic asthma through the influence of the gut microbiota.

## 1. Introduction

Allergic asthma is a chronic inflammatory condition of the airway triggered by allergens. It is estimated that 300 million people worldwide are suffering from asthma, especially children [1]. A predominant T helper type 2 (Th2)-immune response is the classical pathogenesis of allergic asthma. Asthmatic patients mainly rely on the use of β2-agonists, inhaled corticosteroids, leukotriene modifier, anti-IgE therapy and allergen immunotherapy for treatment [2]. However, lifetime dependency, side effects on pediatric patients, and potential unsatisfactory response to these treatments still cause much concerns [2,3,4,5]. Since traditional Chinese medicines (TCM) are increasingly accepted and used as pharmacotherapeutic agents, it may be feasible to develop a novel herbal formula for the treatment of allergic asthma.

Sensitized infants who suffer from atopic dermatitis (AD), a Th2 skin allergic disease, are more likely to develop asthma, implying that AD is one of the predisposing factors of allergic asthma [6]. Therefore, therapeutic intervention on AD is a potential strategy for impeding the onset of asthma [7]. Our previous clinical studies have shown that the quality of life of children who manifested moderate-to-severe AD was significantly improved after treatment with Pentaherbs formula (PHF), which contains TCM concoction of Lonicerae Flos (Jinyinhua), Menthae Herba (Bohe), Moutan Cortex (Danpi), Atractylodis Rhizoma (Cangzhu), and Phellodendri Cortex (Huangbo) at a *w*/*w* ratio of 2:1:2:2:2 [8,9,10,11]. Our in vivo oxazolone-mediated dermatitis mice model revealed that PHF could ameliorate the inflammation of the oxazolone-challenged ears of the mice [12]. We have also reported the anti-inflammatory effects of active ingredients of PHF in IL-31- and IL-33-stimulated eosinophils/dermal fibroblasts co-culture [12]. These active ingredients such as berberine (Ber) and chlorogenic acid (CGA) exhibit anti-allergic and anti-inflammatory activities in rhinitis and asthmatic mice models. [13,14]. Together, it suggested that PHF exhibits anti-inflammatory activities on AD; and could potentially be an alternative adjunct medicine for preventing allergic asthma [12,15,16].

Distinct gut bacterial composition in asthmatic individuals was found to play a vital role in inducing asthma [17]. Changes in lifestyle, disease, use of antibiotics or diet can modify the microbial structure of the gut [18]. Anaerobic polysaccharide-degrading microbes, including species in the phylum Bacteroidetes, ferment and produce metabolic products. These metabolic products subsequently interact with the immune system and protect the host from developing asthma [19,20,21]. Short-chain fatty acids (SCFAs) acetate (C2), propionate (C3) and butyrate (C4) are the major metabolic products produced [22]. Functional receptors for SCFAs and G-protein-coupled receptor 41 and 43 (GPR41 and GPR43) are expressed on immune cells, implicating the potential role of SCFAs on leucocytes activation [23]. Propionate and butyrate regulate the recruitment and the production of cytokines and chemokines of neutrophils and lymphocytes through activating GPR43 and inhibiting histone deacetylase activity [23,24]. Butyrate also induces the differentiation of regulatory T lymphocytes (Treg) and enhances the release of anti-inflammatory cytokine IL-10 [24,25]. Treatments with herbal medicine were also reported to alter the metabolic functions of gut microbes and hence modulate the host immunity [26,27].

To further elucidate the immunomodulatory effects of PHF on allergic asthma, we measured various immunological parameters of the ovalbumin (OVA)-induced allergic asthmatic mice upon PHF treatments. Manipulation of the gut microbiota has been used to control the development of pathological conditions. In view of this, the aim of the current study was to investigate the potential role of microbiota on the immunomodulatory activities of PHF on allergic asthmatic murine model by next-generation sequencing.

## 2. Results

### 2.1. Pentaherbs Formula Reduced Serum OVA-Specific IgE, Airway Hyperresponsiveness (AHR) and Airway Wall Remodeling of OVA-Induced Allergic Asthmatic Mice

Compared to healthy controls, OVA sensitized and challenged mice showed a significantly higher serum OVA-specific IgE (O.D. 0.025 vs. 0.12, respectively, *p* < 0.05) and % change in enhanced pause (Penh) (199% vs. 306%, respectively, *p* < 0.05). Oral intake of PHF (endotoxin = 22.4 EU/mg) for 14 and 8 days, however, reduced the OVA-specific IgE in the serum from O.D. 0.12 to 0.044 and 0.064, respectively (*p* < 0.05). The OVA-specific IgE localized in the lung was barely detectable across groups (data not shown). These results reflect that atopy, the major risk factor of allergic asthma development, of the PHF-treated mice were restrained. Moreover, the % change in Penh of the 14-day PHF-treated mice was decreased from 306 to 225%, implying that the AHR of the mouse was relieved as compared to the non-treated OVA control group. However, the effect of the 8-day PHF oral treatment group was not significant (284%, *p* > 0.05).

**Figure 1:**
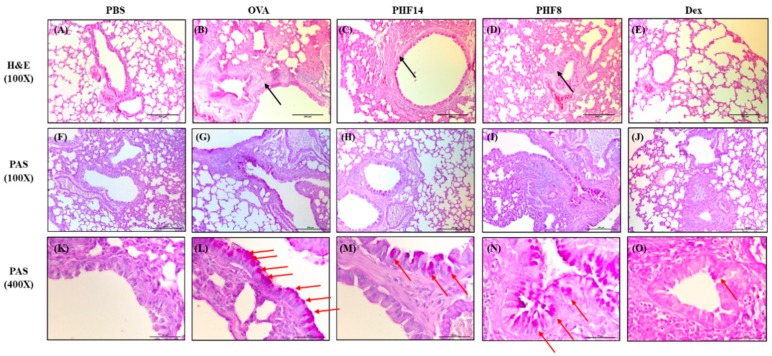
Effects of oral treatment of Pentaherbs formula (PHF) on airway remodeling of the allergic asthmatic mice. (**A**–**E**) Representative H&E staining (100×) and (**F**–**O**) PAS staining (100× & 400×), which denoted the smooth muscle hypertrophy and the goblet cell hyperplasia of the lung, respectively, were performed on day 30 (*n* = 3–4). Black arrows depict the smooth muscle thickness and red arrows denote the distribution of the mucus-secreting goblet cells. PBS: healthy control; OVA: ovalbumin (OVA)-induced allergic asthmatic control; PHF14: mice with 14-day PHF treatment; PHF8: mice with 8-day PHF treatment; Dex: dexamethasone positive control.

Airway wall remodeling is part of the pathogeneses of airway inflammation in allergic asthma. Representative H & E staining of the mouse lung (100×) showed smooth muscle hypertrophy in the airway of OVA control mice (Figure 1B), while the smooth muscle thickness was reduced in the 14-day PHF-treated group as depicted by the black arrows in Figure 1C. Although the thickening of the sub-epithelial basement membrane was not observed across the groups (Figure 1F–J), the distribution of the mucus-secreting goblet cells in the mouse lung, as depicted by the red arrows, demonstrated that goblet cell hyperplasia was improved in the PHF-treated groups as compared to the OVA control group (Figure 1K–O). Differences in angiogenesis across the groups were not observed. PHF treatment could relieve airway remodeling, as summarized in Table 1, however, its effects were not as prominent as the dexamethasone positive control group.

### 2.2. Pentaherbs Formula Significantly Suppressed the Infiltration of Eosinophils and the Release of Allergy-Related Cytokines in BAL of OVA-Induced Allergic Asthmatic Mice

**Figure 2:**
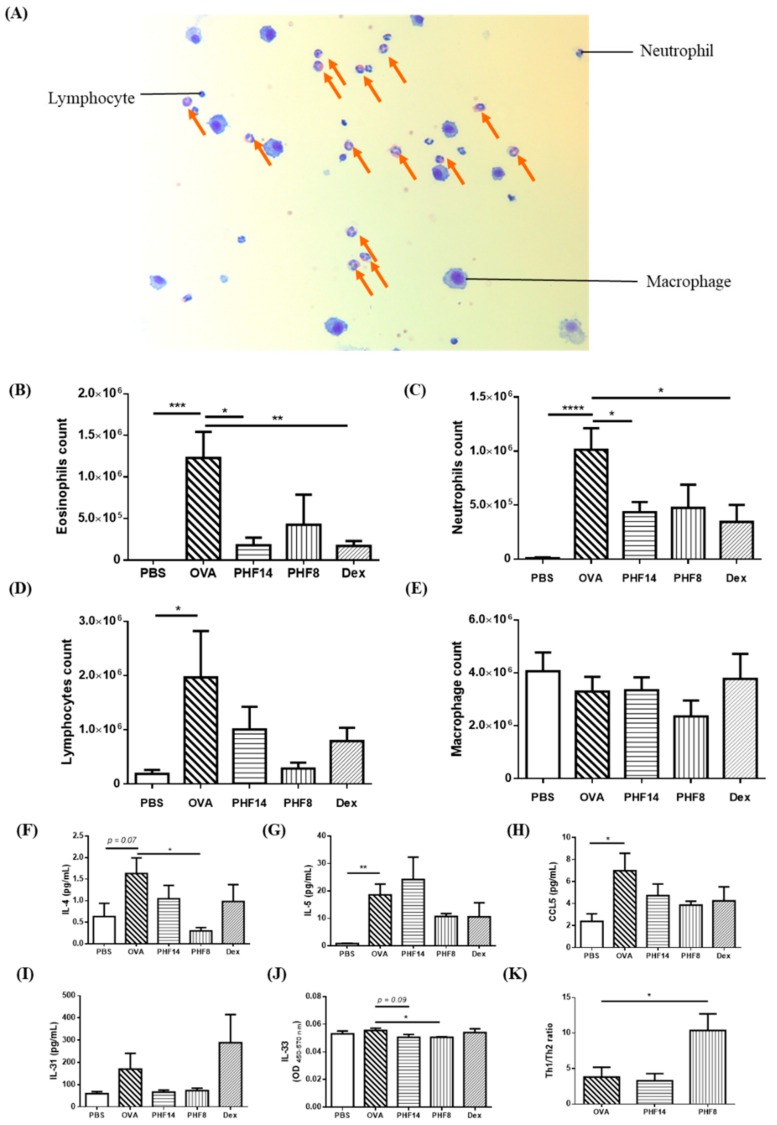
Effects of oral treatment of PHF on the leucocytes, cytokines and chemokines in bronchoalveolar lavage (BAL) of the allergic asthmatic mice. (**A**) Representative eosinophils and other leucocytes in the BAL fluid stained with Shandon Rapid-Chrome Kwik Diff Staining kit (200×). Orange arrows depict the eosinophils presented in the BAL fluid. (**B**–**E**) The total number of eosinophils, neutrophils, lymphocytes, and macrophages were counted and presented in bar charts with means + SEM (*n* = 4–8). (**F**–**H**) The level of IL-4, IL-5 and CCL5 released in the BAL fluid of the mice was measured by Bio-plex pro assay. Results are shown in bar charts with mean + SEM (*n* = 5–8). (**I**,**J**) The level of IL-31 and IL-33 released in the BAL fluid of the mice was measured by enzyme-linked immunosorbent assay (ELISA). Results are shown in bar charts with concentration or O.D. mean + SEM (*n* = 5–8). (**K**) Th1/Th2 ratio, as calculated by dividing the level of Th1 cytokine IFN-γ by Th2 cytokine IL-4 in BAL fluid, was shown in bar chats with mean + SEM (*n* = 5–8). * *p* < 0.05, ** *p* < 0.01, *** *p* < 0.001, **** *p* < 0.0001 when compared with the OVA control group. PBS: healthy control; OVA: OVA-induced allergic asthmatic control; PHF14: mice with 14-day PHF treatment; PHF8: mice with 8-day PHF treatment; Dex: dexamethasone positive control.

An influx of leucocytes, especially eosinophils, into the airway is another pathological characteristic in allergic asthma. Representative Kwik Diff staining showed the presence of leucocytes in the BAL (Figure 2A). In the OVA-induced allergic asthmatic mice, the total numbers of eosinophils, neutrophils, and lymphocytes were significantly increased in the BAL fluid (Figure 2B–D). After treating the OVA-induced mice with PHF orally for 14 days, the total number of the eosinophils and neutrophils were remarkably reduced (all *p <* 0.05). However, the 8-day PHF treatment did not show a prominent modulation in the number of immune cells infiltrating into the lung. These findings were also consistent with the histology of the lung as observed in the H&E staining (400×) (Table 1). The significant changes in these leucocytes in the lung indicate that PHF might ameliorate the allergic inflammation through inhibiting the down-stream Th2 effector lymphocytes.

The significant reduction in eosinophils number at the inflammatory site of the PHF-treated mice prompted us to investigate the activation of Th2 cells. As shown in Figure 2F, the typical Th2 cytokine IL-4 level in BAL fluid was significantly reduced upon 8 days of PHF treatment. As IL-31 is IL-4-dependent [28], in parallel to the reduction in IL-4, the level of IL-31 in the BAL fluid was also decreased (Figure 2I). Both eosinophil activator IL-5 and eosinophils-chemoattractant CCL5 decreased in the BAL fluid of 8-day PHF-treated mice (Figure 2G,H).

Human eosinophils constitutively express functional receptor complex IL-33 receptor ST2; while PHF was found to be effective in suppressing the IL-33-activated eosinophils [29]. Since IL-33 can polarize neutrophils, the key regulatory cells in asthma [30], we found that the eosinophil-activator IL-33 was significantly reduced in the BAL fluid of the 8-day PHF treatment group (*p* < 0.05) (Figure 2J). This is in concordance to our previous findings, whereas the active ingredients of PHF suppressed the release of various pro-inflammatory cytokines from IL-31- and IL-33-stimulated eosinophils [12]. The increase in Th1/Th2 ratio, based on the typical Th1/2 cytokines IFN-γ and IL-4 [31], in the 8-day PHF-treated mice indicates that the Th2-biased immune response was subsided and counteracted by Th1 response (Figure 2K). The level of TGF-β in bronchoalveolar lavage fluid (BALF) was increased from 126 pg/mL to 214 pg/mL upon OVA induction. With the 14-day and 8-day PHF treatments, TGF-β in BALF was reduced to 33.1 pg/mL and 42.5 pg/mL, respectively, although not reaching statistical significance. For IL-13 in BALF, OVA-induced asthmatic mice presented with concentration at 6.82 pg/mL, which was higher than that of the normal control mice (4.94 pg/mL), while 14-day and 8-day PHF treatments could restore the OVA-induced IL-13 in BALF back to 5.40 and 6.44 pg/mL, respectively. The levels of IL-9 and IL-17 in BALF; however, were not detectable across groups (data not shown).

### 2.3. Pentaherbs Formula Altered the Gut Microbial Diversity

**Figure 3:**
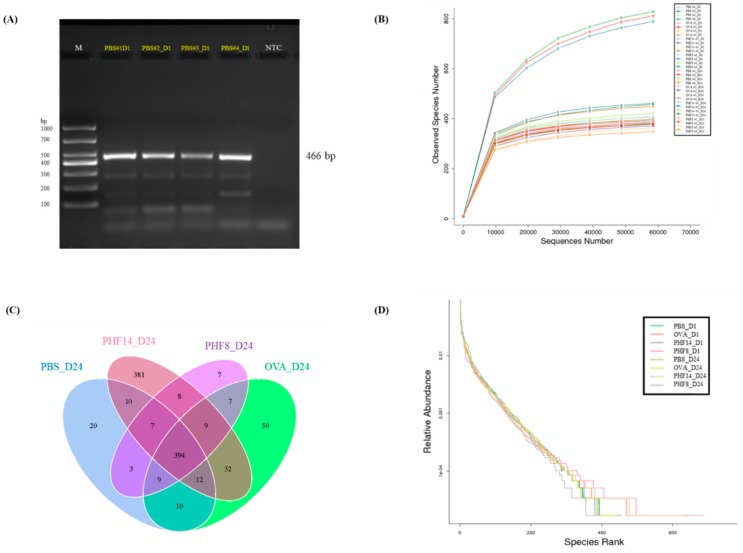
The quality of 16S rRNA gene (V3-4 regions), observed species number in the stool samples, distinct Operational Taxonomic Unit (OTU) observed and disparity in the abundance of each microbial species. Stool samples were collected on day 1 before sensitization and on day 24 before sacrifice. (**A**) Representative cropped gel electrophoresis of the PCR products of 16S rRNA gene (466 base pairs) of stools collected on day 1, as amplified by the 16S rRNA primers. M: DNA ladder DL1000. NTC: negative control. Full-length gel is presented in Supplementary Figure S2. The digital image was captured with a single exposure at 500 ms under UV06 filter and TLUM Mid-Wave light. (**B**) Rarefaction curves of 16S rRNA in stool samples of each mice (#1–4) upon H_2_O, 14-day PHF treatment or 8-day PHF treatment on day 1 and day 24. Observed specie number in each mouse was shown by line chat. (**C**) Venn diagram of shared OTU observed in the stool samples of mice on day 24 (*n* = 4). (**D**) Rank-abundance curve of microbial species in each stool sample (*n* = 4). PBS: healthy control; OVA: OVA-induced allergic asthmatic control; PHF14: mice with 14-day PHF treatment; PHF8: mice with 8-day PHF treatment; D1 and D24: day 1 and day 24, respectively.

The first two pieces of stool sample were collected from each mouse at constant time-points, i.e., upon their arrival on day 1 and 24 h after the last treatment on day 24. A representative gel image shows that a band with molecular size of around 400–500 base pairs was observed in each sample, indicating the presence of the desired V3-V4 amplicon (Figure 3A).

The plateau of the rarefaction curves observed in Figure 3B implies that the sampling depth in identifying new microbial species in each stool sample was sufficiently relative to their overall richness. Hence, the numbers of Operational Taxonomic Unit (OTU) observed are comparable across samples. A total of 3,049,997 raw reads were sequenced. Upon filtering the read sequences with less than 250 base pairs and the chimeric sequences, a total of 2,353,085 reads with an average length of 416 base pairs per sample were included for the following data analysis.

Treatment with PHF altered not only the gut microbial diversity, but also the microbial evenness of the mice. Upon PHF treatment, the total number of OTU observed was increased to 1.22-fold; while the untreated OVA control was reduced to 0.91-fold when compared to day 1. Venn diagram in Figure 3C reveals that a total of 381 distinct OTU were identified upon PHF treatment. These observations indicate that a 14-day PHF treatment increased the bacterial diversity in the gut of the mice, although not statistically significant.

The rank-abundance curve in Figure 3D depicts the relative abundance of microbial species identified in each group on day 1 and 24. The steep declining slopes show that dominating species were found in each group. Among the gut microbes identified on day 24, the remarkably lengthened tail observed in the rank-abundance curve of the 14-day PHF-treated group indicates that the PHF treatment increased the diversity of the non-dominating microbes, making the gut microbiota less even.

The Shannon diversity index (H’) and the Simpson diversity index (D’) denote both the diversity and evenness of a microbial community. H’, the uncertainty in predicting the identity of an individual, is higher in a highly diverse community when compared to a less diverse community [32]. D’, on the other hand, is the probability of picking two individuals that belong to two different species; and its value also increases with the diversity [32]. In our study, both indices were increased in all PHF-treated groups on day 24 (Table 2). Notably, the Shannon diversity index was higher in the PHF-treated group, when compared to the OVA control mice.

### 2.4. Effects of Pentaherbs Formula on the Change in Abundance at Phylum and Genus Levels.

**Figure 4:**
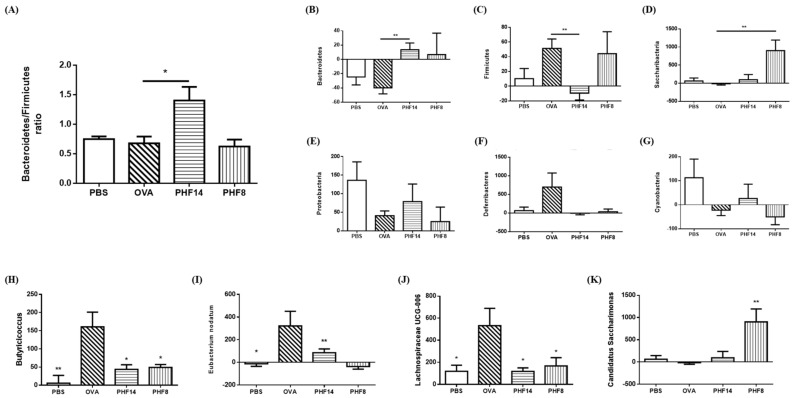
Effects of PHF treatment on gut microbial community structure at the phylum and genus levels. Stool samples were collected on day 1 before sensitization and on day 24 before sacrifice. (**A**) Relative Bacteroides/Firmicutes ratio in stool samples on day 24. (**B**–**G**) Percentage change in absolute abundance in the phyla of Bacteroidetes, Firmicutes, Saccharibacteria, Proteobacteria, Deferribacteres, and Cyanobacteria, respectively. (**H**–**K**) Percentage change in absolute abundance in the genera of Butyricicoccus, Eubacterium nodatum, Lachnospiraceae UCG-006 and Candidatus Saccharimonas, respectively. Results are shown in bar charts with mean + SEM. (*n* = 4). * *p* < 0.05, ** *p* < 0.01 when compared with the OVA control group. PBS: healthy control; OVA: OVA-induced allergic asthmatic control; PHF14: mice with 14-day PHF treatment; PHF8: mice with 8-day PHF treatment; D1 and D24: day 1 and day 24, respectively.

PHF treatment induced changes in the overall microbial community structure in the gut. At the phylum level, the gut of the OVA control mice was dominated by butyrate-producing Firmicutes on day 24 (Figure 4A). However, upon PHF treatment for 14 days, the Bacteroidetes became the dominating group in the gut, as revealed by the significant increase in Bacteroidetes/Firmicutes ratio (*p* < 0.05, Figure 4A). Of note, the relative abundance of Bacteroidetes (Figure 4B) and Saccharibacteria (Figure 4D) were significantly increased in the PHF-treated mice for 14 days and 8 days, respectively, when compared to the OVA control (all *p* < 0.01). The abundance of Firmicutes, however, was significantly reduced upon the 14-day PHF treatment (Figure 4C). Upon 14-day PHF treatment, the other major phyla, including the Proteobacteria, Deferribacteres and Cyanobacteria, each showed a restoring trend in their relative abundance to the level comparable to their healthy counterparts, although not statistically significant (Figure 4E–G).

At the genus level, the abundance of Butyricicoccus, Eubacterium nodatum and Lachnospiraceae UCG-006, all under the Firmicutes phylum, were generally increased in the gut of the OVA-induced allergic asthmatic mice (Figure 4H–J, all *p* < 0.05). Upon PHF treatment, their increase in abundance is significantly reduced (all *p* < 0.05); and becomes comparable to the change observed in the healthy controls. Moreover, the number of Candidatus Saccharimonas, a genus under the Saccharibacteria phylum, in the gut of the mice was significantly enriched upon 8-day PHF treatment (all *p* < 0.05, Figure 4K and Table 3). The subtle changes in the abundance of other predominating microbes, mainly from the phyla Bacteriodetes, Deferribacteres, Firmicutes, Proteobacteria, and Saccharibacteria, all indicate that PHF treatment helped the restoration of the microbial abundance to a level comparable to the healthy control (Table 3). The above results recapitulate that the abundance of several genera in the phylum of Firmicutes and Saccharibacteria were significantly altered by the PHF treatment.

### 2.5. Changes in Short-Chain Fatty Acids in the Gut, Modulation on Splenic Treg Cells Population and Serum IL-10 Level

**Figure 5:**
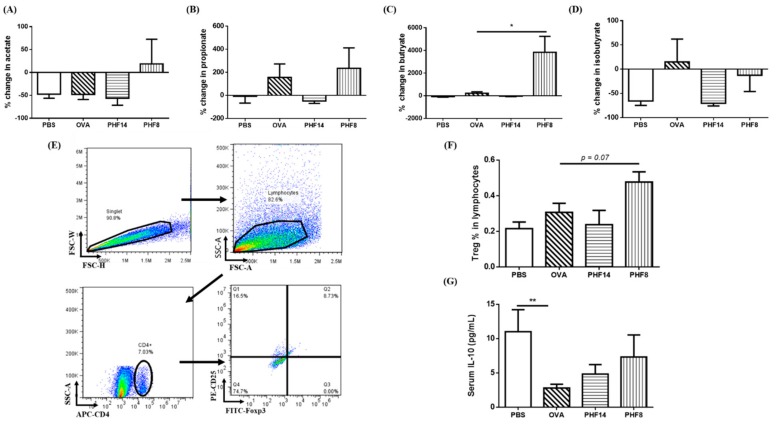
Effects of PHF treatment on short chain fatty acids (SCFAs) content in stool, modulation on the CD4^+^CD25^high^Foxp3^+^ Treg cells population and systemic IL-10 level of allergic asthmatic mice. Stool samples were collected on day 1 before sensitization and on day 24 before sacrifice. GC-MS quantification was performed to evaluate the concentration of SCFAs in the stool samples. Percentage changes of (**A**) acetate, (**B**) propionate, (**C**) butyrate and (**D**) isobutyrate between day 1 and day 24 are shown in bar charts with mean + SEM. (*n* = 3–5). (**E**) Gating strategy of the splenic CD4^+^CD25^high^Foxp3^+^ Treg cells was shown by representative dot-plots using flow cytometry. (**F**) Population of splenic CD4^+^CD25^high^Foxp3^+^ Treg cells in the lymphocytes was determined by flow cytometry. (**G**) The serum level of IL-10 was measured by Bio-plex pro assay. Results were shown in bar charts with mean + SEM (*n* = 4–8). * *p* < 0.05, when compared with the OVA control group. PBS: healthy control; OVA: OVA-induced allergic asthmatic control; PHF14: mice with 14-day PHF treatment; PHF8: mice with 8-day PHF treatment.

Acetate, propionate, and butyrate are the most abundant short-chain fatty acids found in the gut [22]. By calibrating the SCFA standards using the peak areas of the total ion current (TIC), SCFAs and its percentage changes were quantified (Figure 5A–C). The amount of acetate, the major SCFAs, was increased upon 8-day PHF-treatment (Figure 5A). The amount of Treg cells-inducing butyrate was significantly increased in mice upon 8-day PHF treatment (Figure 5C), despite the significant decrease in the relative abundance of the butyrate-producing Odoribacter (Table 3). The concentration of propionate in the 14-day PHF-treated mice was found to be decreased, with a trend similar to the healthy controls (Figure 5B). The concentration of isobutyrate, the isomer of butyrate with a branched methyl group, was also decreased in the PHF-treated groups (Figure 5D).

While Treg cells actively protect healthy non-atopic individuals from Th2 responses against allergens, they resolve OVA-induced inflammatory responses in murine models [33]. In the present study, the percentage of splenic fully differentiated CD4^+^CD25^high^Foxp3^+^ Treg cells in the lymphocytes population was increased (*p* < 0.07) upon 8-day PHF treatment, when compared with the OVA asthmatic control (Figure 5F).

The enhancement in Treg cells function in the PHF-treated mice could be reflected by the moderate up-regulation of anti-inflammatory cytokine IL-10 level in the serum (Figure 5G). IL-10 are secreted by Treg cells or antigen-presenting cells, with inhibitory activities on Th1 cytokine production and Th2 responses [34]. The above results indicated the potential immune-regulatory effects of PHF on Treg cells and their functions. The findings were also in line with our microbiota results, as gut microbiota plays a vital role in the development of in vivo immune tolerance in allergic asthma [35].

## 3. Discussion

Allergic asthma is the major subtype of the asthma, accounting for 70–80% of total cases [36]. Th2 cell-mediated immune response leading to pulmonary eosinophilia remains the most representative pathogenesis among asthmatic patients. Several epidemiological studies have indicated that extrinsic AD has a causal link to allergic asthma development [7]. We have previously shown that PHF could ameliorate inflammatory symptoms and eosinophil infiltration in oxazolone-induced dermatitis-like murine model [12]. The anti-inflammatory actions of PHF are multi-targeted [10,15,16]. Its active components such as CGA (1.20%), gallic acid (0.479%) and Ber (0.022%) also showed anti-inflammatory activities on allergic inflammation-related IL-31- and IL-33-stimulated in vitro models [12]. Kim et al. have also reported that CGA, the major pure substance in PHF, exhibits suppressive effect in pulmonary eosinophil infiltration, OVA-specific IgE and Th2-related cytokine production in OVA-induced allergic asthmatic mice [14]. Therefore, in the current study, a well-established ovalbumin (OVA)-induced allergic asthmatic mice model, with both clinical and pathophysiological features of allergic asthma in human, was used to further investigate the anti-inflammatory action of PHF [37]. We showed that oral treatment of PHF relieved the inflammatory conditions in OVA-induced allergic asthmatic mice and altered the gut microbiome and content of SCFAs propionate and butyrate. This study thus provided novel scientific evidence to support that PHF may potentially prevent the development of subsequent atopic disorders.

In our current study, OVA was injected into the mice intraperitoneally to provide a systemic immunization prior to the airway challenge. Mice were either given PHF before sensitization began, for a total of 14 days; or given PHF five days before the challenge, for a total of 8 days. Distinct immunomodulatory activities were also observed in these two PHF treatment groups. A shorter and latter intervention might modulate the upstream participants in the pathogenesis of allergic asthma, as evidenced by the increase in splenic Treg cells, an increase in Th1/Th2 ratio, a decrease in asthma-related BALF IL-13 level and the significant decrease in IL-4 and IL-33 levels in the BAL fluid of the 8-day PHF-treated mice; while a longer and earlier PHF-intervention relieve the inflammatory symptoms, including the reduction in airway remodeling and the marked decrease in leucocyte infiltration in the lung of the 14-day PHF-treated mice. These changes might contribute to the reduction of eosinophil number in the inflammatory site and hence improve the subsequent smooth muscle remodeling and airway hyperresponsiveness. Our results therefore indicated that PHF has a potential in preventing the development of Th2-cell mediated allergic asthma.

Infants with AD and allergic asthma were found to have smaller microbial diversity than their healthy counterparts [38,39]. The exposure to a broader diversity of microbes by the host at early stage facilitates the recognition of dangerous organisms, maintains the activation of innate immune system and establishes immune tolerance against harmless antigens [40]. Based on the total OTU observed and the Shannon diversity index, the microbial diversity in the gut of 14-day PHF-treated mice was higher than that of the allergic asthmatic controls (Table 2). In the current study, the PHF water extract mainly contains soluble carbohydrates (65.9%), proteins (9.4%), soluble fibers (6.2%), and a minute amount of insoluble dietary fibers (0.2%) because the insoluble polysaccharides were filtered during the extraction process. It is postulated that certain microbes that favors the use of these soluble substrates would achieve a more optimal energy harvest under the PHF treatment.

In concordance to the previous findings in asthma-protective mice upon high-fiber consumption [20], significant increase in the abundance of the gram-negative Bacteroidetes was found in our 14-day PHF-treated mice (Figure 4B). Bacteroidetes are the most abundant bacteria in the anaerobic environment of human large intestine, and they utilize small and soluble carbohydrates, e.g., starch, as energy sources [41]. Metabolic products acetate and propionate are mainly produced by Bacteroidetes. These SCFAs influence the dendritic cell hematopoiesis and impair their activation on Th2 effector cells in lung [20]. Dendritic cells not only inhibit allergic responses by enhancing Th1 responses, but are also involved in immuno-regulation through Treg cells [42]. Firmicutes, on the other hand, primarily produce butyrate as their metabolic end products [22]. A substantial decrease in abundance of Firmicutes in the gut was observed upon a 14-day PHF treatment; although the change in butyrate level was insignificant (Figure 4C and Figure 5C). A previous study has found that a low-fiber consumption accounts for the reduction in acetate and butyrate [19,20]. Increase in butyrate may further lead to the reduction of eosinophilic airway inflammation [21]. Our study shows that the 14-day PHF-treated mice have a significantly reduced number of eosinophils infiltrated to the lung, despite that the butyrate content in their stool samples were not reduced.

PHF-treatment after OVA sensitization showed a prominent outgrowth in the Candidatus Saccharimonas (Figure 4K), which are lactate and acetate producers. They are associated with inflammatory mucosal disease; and may modulate immune response through repressing TNF-α gene expression in macrophage [43]. The exclusive expansion of Candidatus Saccharimonas in the 8-day PHF-treated mice indicates that this strain of microbe might play a protective role in the development of allergic asthma in sensitized individuals. Besides, the enrichment of Firmicutes in the 8-day PHF treated-mice was also coherent with the significant increase in the butyrate level in their stool samples. Butyrate could exhibit anti-inflammatory activities through the suppression of NF-κB signaling by preventing the degradation of proteasomal IκB and modulation of NF-κB transcription through inhibiting the histone deacetylase (HDAC) [24]. Furthermore, the concentration of propionate in the stool samples of the 8-day PHF-treated mice was increased when compared to day 1. Propionate is an anti-inflammatory mediator to promote the release of IL-10, the anti-inflammatory cytokine in Treg cells-mediated suppression, in inflammatory diseases [24]. The generation of Foxp3-expressing Treg cells in the 8-day PHF-treated mice was probably mediated by the SCFAs produced from the commensal microbes [25]. Since the alteration in the microbiota in both PHF treatment groups generally resembled changes found in the healthy control, our study provides evidence that PHF modulated the composition of microbiota and the production of immunomodulatory SCFAs. Restoration of non-pathogenic microbes and alleviation in the inflammatory symptoms and biomarkers were observed in both PHF treatment groups. In view of the “Old Friends Hypothesis”, which denotes the establishment of immunoregulation by early exposure to co-evolved microbes, including the commensal microbes, could protect individuals from allergic diseases [40]; the alteration in intestinal commensal microbes identified in this study might shed lights on the immunomodulatory mechanisms of PHF.

The striking difference in certain microbial abundance and the SCFAs contents between the two treatment groups suggested that the PHF effects on microbiota and SCFAs might be rather transient. Besides, the treatment periods could be a critical factor in the PHF efficacy for preventing allergic asthma. It is speculated that the difference in microbiota and SCFA alteration might be responsible for the differences in immunological and histological features as described above.

A limitation of the current study is the lack of data showing the actual SCFAs metabolism in the gut. The concentration of SCFAs could depend on the participation of other microbes through cross-feeding, which leads to the generation of new SCFAs in the consumption of SCFAs produced by other microbes [44]. Moreover, SCFAs are also readily absorbed and utilized by colonocytes as their major energy source, and only 5% of the SCFAs will be secreted in the stool [22]. Thus, the actual intestinal SCFAs metabolism capacity of microbes might be underestimated in the current study. Enrichment of butyryl-CoA, acetate CoA-transferase gene encoded the enzyme in the last step of butyrate production, in gut microbiota may be further explored to elucidate the colonic butyrate production in gut microbiota [44]. The immuno-protective role of particular microbial strains in the phylum of Bacteroidetes, Firmicutes, and Saccharibacteria should also be confirmed by the adoptive transfer of gut microbes from the PHF-treated group to untreated OVA controls. Previous study has shown that CXCR5^+^CD4^+^ follicular T helper cells (Tfh) and Foxp3^+^ follicular regulatory T cells (Tfr) for the regulation of B cell isotype switching and affinity maturation in germinal centers, can induce IgE production in allergic asthma, thereby playing crucial roles in the exacerbation of allergic asthma [45]. Moreover, Tfh cells have been shown to interact with the gut microbiota for the maintenance of mucosal barriers of the gut [46]. Tfh and Tfr cells also play important roles in the dynamic diversification of gut microbiota by regulating IgA responses to gut microbiota, for maintaining appropriate gut microbial communities and immune homeostasis [47,48]. As a result, the modulation of gut microbiota by PHF may subsequently lead to the immune-modulation of Tfh and Tfr cells, as evidenced by the increased serum IL-10 (Figure 5G), to suppress the allergic inflammation in allergic asthma. However, whether PHF can directly modulate the activities of Tfh and Tfr cells in allergic asthma needs further investigation.

## 4. Materials and Methods

### 4.1. Preparation of Pentaherbs Formula (PHF)

The five herbal components of PHF at a *w*/*w* composition of 50 g Lonicerae Flos (Jinyinhua), 25 g Menthae Herba (Bohe), 50 g Moutan Cortex (Danpi), 50 g Atractylodis Rhizoma (Cangzhu), and 50 g Phellodendri Cortex (Huangbo) were purchased, authenticated and extracted by hot water extraction method as previously described [12]. Briefly, the mixture of the five herbal components was refluxed in boiling water at 100 °C for 1 h. Extraction was repeated twice to obtain the water crude extract. The filtered extract was freeze-dried into powder and then dissolved in distilled water to obtain the final concentration (46 mg/mL). The endotoxin level of the PHF water extract was quantified by PyroGene Recombinant Factor C Endotoxin Detection Assay (LONZA Group, Basel, Switzerland).

### 4.2. Animal Experiment

Inbred adult male BALB/c mice (8-week-old, 20 g body weight) were purchased from The Laboratory Animal Services Centre, The Chinese University of Hong Kong (Hong Kong, China). All animals were given normal extruded diet (Envigo, Somerset, NJ, USA). All the experiments were approved by the Department of Health of The Government of the Hong Kong Special Administrative Region; and the Animal Experimentation Ethics Committee of The Chinese University of Hong Kong.

Ovalbumin (OVA)-induced allergic asthmatic mice model was established as previously described [37]. Briefly, 1% ovalbumin (Sigma-Aldrich, Saint Louis, MO, USA) in Imject Alum Adjuvant (Thermo Fisher Scientific, Rockford, IL, USA) were injected to the mice by intraperitoneal (i.p.) injection on day 1 and 8. The mice were challenged with nebulized 10 mg/mL OVA twice a day from day 21 to 23. H_2_O (0.2 mL) or PHF (9.2 mg) in 0.2 mL H_2_O were orally administered to the mice from day 1 for 14 days or from day 16 for 8 days. Dexamethasone (100 µg/mL) (Sigma-Aldrich) was administered to the mice as positive control on day 1, day 8, and from day 21 to 23. On day 24, mice were euthanized after bronchoconstriction challenge. Stool samples were collected under pathogen-free conditions before the first allergen sensitization on day 1 and before the bronchoconstriction challenge on day 24. Sera were collected for the determination of cytokines and IgE levels.

### 4.3. Measurement of Lung Function by Bronchoconstriction Challenge

On day 24, the airway hyper-responsiveness of all mice against methacholine was tested using a FinePointe for Whole Body Plethysmography (Data Sciences International, New Brighton, MN, USA). The degree of bronchial constriction of the mice triggered by acetyl-methylcholine chloride (6.25 mg/mL, Sigma-Aldrich) challenge were measured and recorded as “enhanced pause” (Penh). The percentage (%) change in Penh normalized to baseline level of each group was calculated.

### 4.4. Histological Examination

The lungs were fixed in 4% paraformaldehyde and embedded in paraffin. Paraffin sections (6 µm) of mouse lung tissues were then stained with Hematoxylin and Eosin (H&E) (Beyotime Institute of Biotechnology, Haimen, China) for standard histopathological examination. Mucus secretions were stained using the Periodic Acid-Schiff (PAS) staining kit (Sigma-Aldrich) for the determination of goblet cells hyperplasia. The degree of airway remodeling was semi-quantitively graded as normal (-), mild (+), moderate (++) or severe (+++) based on the smooth muscle thickening, goblet cell hyperplasia and eosinophil infiltration. Briefly, smooth muscle thickness in the airway was measured by software ImageJ (U.S. National Institutes of Health, Bethesda, MD, USA). The percentage of goblet cells and the number of infiltrated eosinophils were counted as previously described [12,49].

### 4.5. Bronchoalveolar Lavage (BAL) Fluid Collection and Immune Cell Determination

BAL fluid of the mice was collected by instilling 0.9 mL of cold PBS with 2 mM EDTA and 2% fetal bovine serum (FBS) into the lung of the mice once the mice were sacrificed. The supernatant of BAL fluid was collected for the measurement of cytokines and IgE levels. The cells in the BAL fluid were counted and stained using the Kwik Diff Staining kit (Thermo Fisher Scientific). The number of eosinophils, neutrophils, lymphocytes, and macrophages in the BAL fluid per mL were counted in three random fields per specimen at 200× magnification using Leica DM6000B microscope (Leica Microsystems GmbH, Wetzlar, Germany) and the Leica Application Suite software (Leica Microsystems GmbH).

### 4.6. Measurement of OVA-Specific IgE, Total IgE, Cytokines and Chemokines in Serum and BAL Fluid

Concentrations of murine Th1 cytokines interferon (IFN)-γ, Th2 cytokines IL-4, IL-5, IL-13 and RANTES/CCL5, pro-inflammatory cytokine IL-17 and anti-inflammatory cytokine IL-10 in serum and BAL fluid were determined using Multiplex MAP kit (EMD Millipore Corp., Billerica, MA, USA) and Bio-plex pro assay kit reagents using Luminex Bio-plex 200 suspension array system (Bio-Rad Corp. Hercules, CA, USA). The levels of IL-31 and IL-33 in BAL fluid of the mice were measured by enzyme-linked immunosorbent assay (ELISA) kits (Thermo Fisher Scientific and BioLegend, San Diego, CA, USA, respectively). Th1/Th2 ratio was calculated based on the Th1 IFN-γ and Th2 IL-4 levels in BAL fluid. Murine OVA-specific IgE, total IgE, IL-9 and TGF-β concentrations in the serum and BAL fluid were determined using ELISA kits (BioLegend).

### 4.7. Determination of Splenic Treg Cells

The excised spleens of the mice were passed through a 70 µm cell strainer to obtain single cell suspension in PBS with 2 mM EDTA & 2% FBS. Red blood cells were lysed, and mononuclear cells were washed and re-suspended in RPMI supplemented with 10% FBS. Percentages of CD25^high^Foxp3^+^ Treg cells in lymphocytes were determined using Mouse Treg Flow Kit (FOXP3 Alexa Fluor 488/CD4 APC/CD25 PE, BioLegend) with flow cytometer BD FACSVia (BD Biosciences, San Jose, CA, USA).

### 4.8. Determination of Gut Microbiota

Bacterial 16S rRNA gene in the stool samples were extracted using TIANamp Stool DNA Extraction Kit (TIANGEN Biotech, Beijing, China) with QIAGEN Ribonuclease A (QIAGEN, Hilden, Germany). Concentration and purity of the extracted DNA were measured using NanoDrop 1000 (Thermo Fisher Scientific); and amplified using primers (341F primer: 5′-CCTAYGGGRBGCASCAG-3′; 806R primer: 5′-GGACTACHVGGGTWTCTAAT-3′) which target the hypervariable regions V3-V4 of bacterial 16S rRNA gene [50,51]. Amplicon sequencing library was constructed, and sequencing was performed using Illumina HiSeq 2500 (Illumina, San Diego, CA, USA). Quantitative Insights Into Microbial Ecology (QIIME) pipeline (version 1.7.0) and UCHIME algorithm was used to filter data, and detect and remove chimeric sequences, respectively. Sequences with >97% similarity were assigned as the same Operational Taxonomic Unit (OTU) by the OTU clustering software package UPARSE (version 7.0.1001). The software Mothur and SSU datasets of the SILVA rRNA Database were employed for species annotation with confidence threshold of 0.8. Sequencing data analysis on the alpha and beta diversity were done by QIIME and R (version 2.15.3).

### 4.9. Determination of SCFAs in Stools

Acetic acid, propionic acid, butyric acid, and its isomer isobutyric acid in stool samples of the mice were detected by Gas Chromatograph Mass spectrometer-QP2010 (GC-MS) (Shimadzu, Tokyo, Japan). Each stool sample in methanol (50 mg/mL) was homogenized by vigorous vortexing for 10 s and ultrasound sonication for 10 min. The mixture was then centrifuged at 14,000 rpm for 5 min at room temperature. The supernatant was further diluted with methanol by 10-fold and 1 µL of sample was vaporized at 230 °C upon injection. An Agilent J&W fused silica capillary column DB-FFAP (Agilent, Santa Clara, CA, USA) was used to separate the compounds. Samples were then ionized by electron impact at −70 eV at 200 °C, and analyzed by Quadrupole mass spectrometer. GCMSsolution Software (Shimadzu, Japan) was employed to identify each SCFA based on their corresponding mass spectra and retention times; and to quantify concentrations of the SCFAs by the peak areas of the total ion current (TIC) with reference to the calibration curve of the standards (Supplementary Figure S1) [52].

### 4.10. Data Analysis

All experiments were performed at least in triplicate. Results were analyzed using multiple Student’s *t*-test for comparisons using GraphPad PRISM software version 6.0 (GraphPad Software, San Diego, CA, USA). A probability (*p*) < 0.05 was considered significantly different.

## 5. Conclusions

This study demonstrated that oral administration of PHF exhibits significant improvement on inflammatory symptoms and ameliorates the release of allergy-related cytokines, with alteration in the gut microbiome and metabolite content in OVA-induced allergic asthmatic mice model. Our work further extends the findings of other groups by showing that intake of herbal extracts, which contain only trace amount of insoluble dietary fiber, possesses potential protective effects in the development of allergic asthma. Our findings also shed lights on the biochemical mechanisms and the use of herbal medicine, which could ameliorate symptoms of atopic dermatitis, for the prevention of subsequent development of allergic asthma.

## Figures and Tables

**Table 1:** Degree of airway remodeling of the mice.

	PBS	OVA	PHF14	PHF8	Dex
Smooth muscle hypertrophy	-	++	-	++	+
Goblet cell hyperplasia	-	+++	+	-	+
Eosinophil infiltration	-	+++	+	+++	-

PBS, healthy control; OVA, OVA-induced allergic asthmatic control; PHF14, asthmatic mice with 14-day PHF treatment; PHF8, asthmatic mice with 8-day PHF treatment; Dex, asthmatic mice treated with dexamethasone; -, normal; +, mild; ++, moderate; +++, severe.

**Table 2:** OTU richness, diversity and evenness of total microbiota identified in stool samples of allergic asthmatic mice on day 1 and day 24.

	Day 1				Day 24			
	PBS	OVA	PHF14	PHF8	PBS	OVA	PHF14	PHF8
Mean of total OTU observed	413	490	398	525	394	400	485	370
Mean of Shannon diversity index	6.49	6.29	6.30	6.21	6.36	6.32	6.44	6.48
Mean of Simpson index	0.975	0.968	0.965	0.943	0.970	0.964	0.972	0.979

PBS: healthy control; OVA: OVA-induced allergic asthmatic control; PHF14: mice with 14-day PHF treatment; PHF8: mice with 8-day PHF treatment.

**Table 3:** Mean of relative abundance of predominating microbes (with relative abundance > 1% at any sample) at the genus level in stool samples on day 1 and day 24. *p*-values of paired t-test refer to comparison against the relative abundance on day 1.

Taxonomy		Mean of Relative Abundance (%)								*p*-Value			
		Day 1				Day 24							
Phylum	Genus	PBS	OVA	PHF 14	PHF8	PBS	OVA	PHF 14	PHF8	PBS	OVA	PHF 14	PHF 8
Bacteroidetes	Alistipes	9.26	8.24	7.66	11.2	8.50	8.10	7.59	5.75	0.17	0.95	0.99	0.19
	Bacteroides	4.48	1.46	1.26	1.54	3.29	4.57	3.85	2.26	0.78	0.09	0.13	0.64
	Odoribacter	1.47	1.96	0.86	1.53	1.24	0.85	0.49	0.80	0.38	0.22	0.13	**0.03**
	Rikenella	1.62	2.17	1.54	1.11	3.23	0.96	0.59	0.39	0.29	0.09	**0.02**	0.061
	Rikenellaceae_RC9_gut_group	1.10	0.86	0.60	0.88	0.68	1.48	0.46	0.18	0.31	0.39	0.42	0.08
Deferribacteres	Mucispirillum	0.13	0.07	0.80	0.34	0.19	0.30	0.11	0.24	0.58	**0.04**	0.39	0.59
Firmicutes	[Eubacterium]_xylanophilum_group	1.21	0.50	0.67	0.37	0.62	1.09	0.52	0.76	0.54	**0.01**	0.73	0.25
	Lachnospiraceae_NK4A136_group	14.9	12.5	13.2	11.2	20.0	19.8	10.1	20.5	0.16	0.32	0.56	0.17
	Lactobacillus	2.16	2.40	8.22	12.3	3.16	4.30	2.13	1.87	0.31	0.35	0.17	0.30
	Ruminiclostridium	1.83	1.14	1.13	1.30	0.65	2.01	0.82	1.00	**0.04**	0.26	0.29	0.65
	Ruminiclostridium_9	1.84	1.36	1.47	1.70	1.21	1.64	1.34	2.03	0.08	0.58	0.57	0.51
	Ruminococcaceae_UCG-014	5.49	2.51	5.08	5.55	7.63	3.99	6.80	4.89	0.65	0.40	0.68	0.85
	unidentified_Lachnospiraceae	1.83	1.03	1.76	1.78	1.89	2.73	2.20	3.43	0.79	0.07	0.52	0.13
	unidentified_Ruminococcaceae	0.98	0.89	0.81	0.70	0.45	0.84	0.60	1.70	0.21	0.86	0.13	0.14
Proteobacteria	Desulfovibrio	0.59	0.81	0.76	0.51	2.74	1.58	2.06	2.02	0.11	0.32	0.11	0.06
Saccharibacteria	Candidatus_Saccharimonas	1.00	2.60	1.96	0.26	0.98	1.55	1.66	1.64	0.94	0.35	0.70	**0.01**

PBS: healthy control; OVA: OVA-induced allergic asthmatic control; PHF14: mice with 14-day PHF treatment; PHF8: mice with 8-day PHF treatment.

## Supplementary Materials

Supplementary Figures S1 and S2 are available online.
