# Supplementary material for: Anti-Inflammatory Activities of Pentaherbs formula and Its Influence on Gut Microbiota in Allergic Asthma
Source: Molecules. 2018 Oct 26;23(11):2776. doi: 10.3390/molecules23112776 (PMC6278535; doi:10.3390/molecules23112776)
Supplement: Supplementary file 1 [file molecules-23-02776-s001.pdf]

Anti-inflammatory activities of Pentaherbs formula and its influences on gut microbiota in allergic asthma

Miranda Sin-Man Tsang[1], Sau Wan Cheng[1], Jing Zhu[2], Karam Atli[2], Ben Chung Lap Chan[1], Dehua Liu[1], Helen Yau-Tsz Chan[2], Xiaoyu Sun[2], Ida Miu-Ting Chu[2], Kam-Lun Hon[3], Christopher Wai-Kei Lam[4], Pang-Chui Shaw[1,5,6], Ping Chung Leung[1], Chun-Kwok Wong[1,2,6*]

[1]Institute of Chinese Medicine and State Key Laboratory of Phytochemistry and Plant Resources in West China, The Chinese University of Hong Kong, Hong Kong; [2]Department of Chemical Pathology, The Chinese University of Hong Kong, Prince of Wales Hospital, Hong Kong; [3]Department of Paediatrics, The Chinese University of Hong Kong, Prince of Wales Hospital, Hong Kong; [4]State Key Laboratory of Quality Research in Chinese Medicines, Macau Institute for Applied Research in Medicine and Health, Macau University of Science and Technology, Macau; [5]School of Life Sciences, The Chinese University of Hong Kong, Hong Kong; [6]Li Dak Sum Yip Yio Chin R & D Centre for Chinese Medicine, The Chinese University of Hong Kong, Hong Kong.

Running title: Microbiota in allergic asthma

*Correspondence: Professor Chun Kwok Wong, Department of Chemical Pathology, The Chinese University of Hong Kong, Prince of Wales Hospital, Shatin, N.T., Hong Kong. Tel: (852) 3505 2964, Fax: (852) 2636 5090, E-Mail: ck-wong@cuhk.edu.hk

Supplementary Fig. S1

(A)

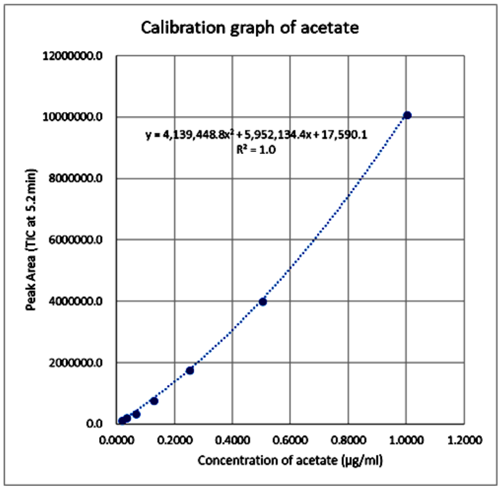

(B)

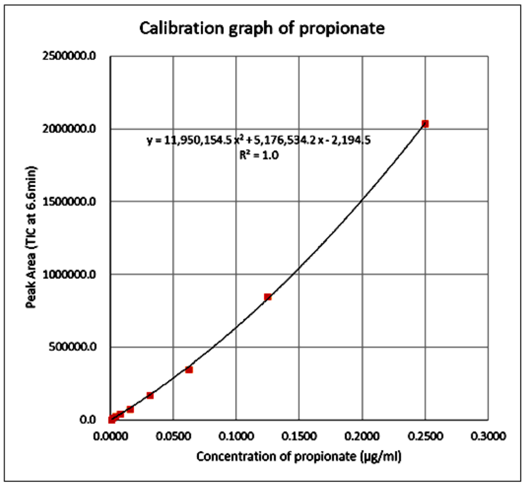

(C)

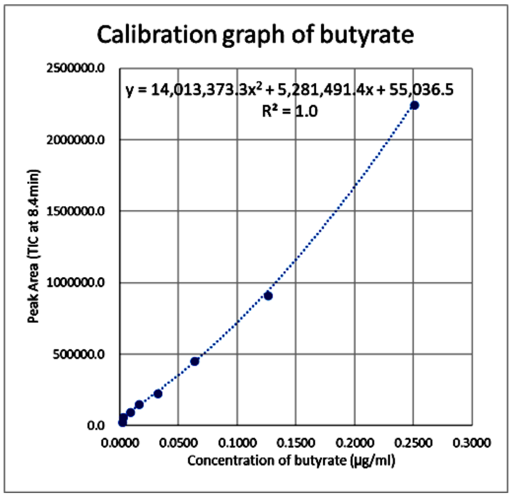

(D)

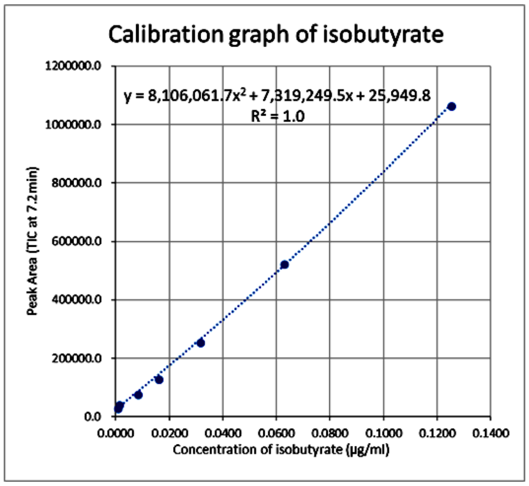

Supplementary Fig. S1. Calibration curve of each SCFA standard. Peaks of (A) acetate, (B) propionate, (C) butyrate and (D) isobutyrate were identified according to the mass spectra and retention times, which were 5.2, 7.6, 8.5 and 7.2 minutes, respectively. Concentration of each SCFA in the stool sample was calculated from the calibration curves.

Supplementary Fig. S2

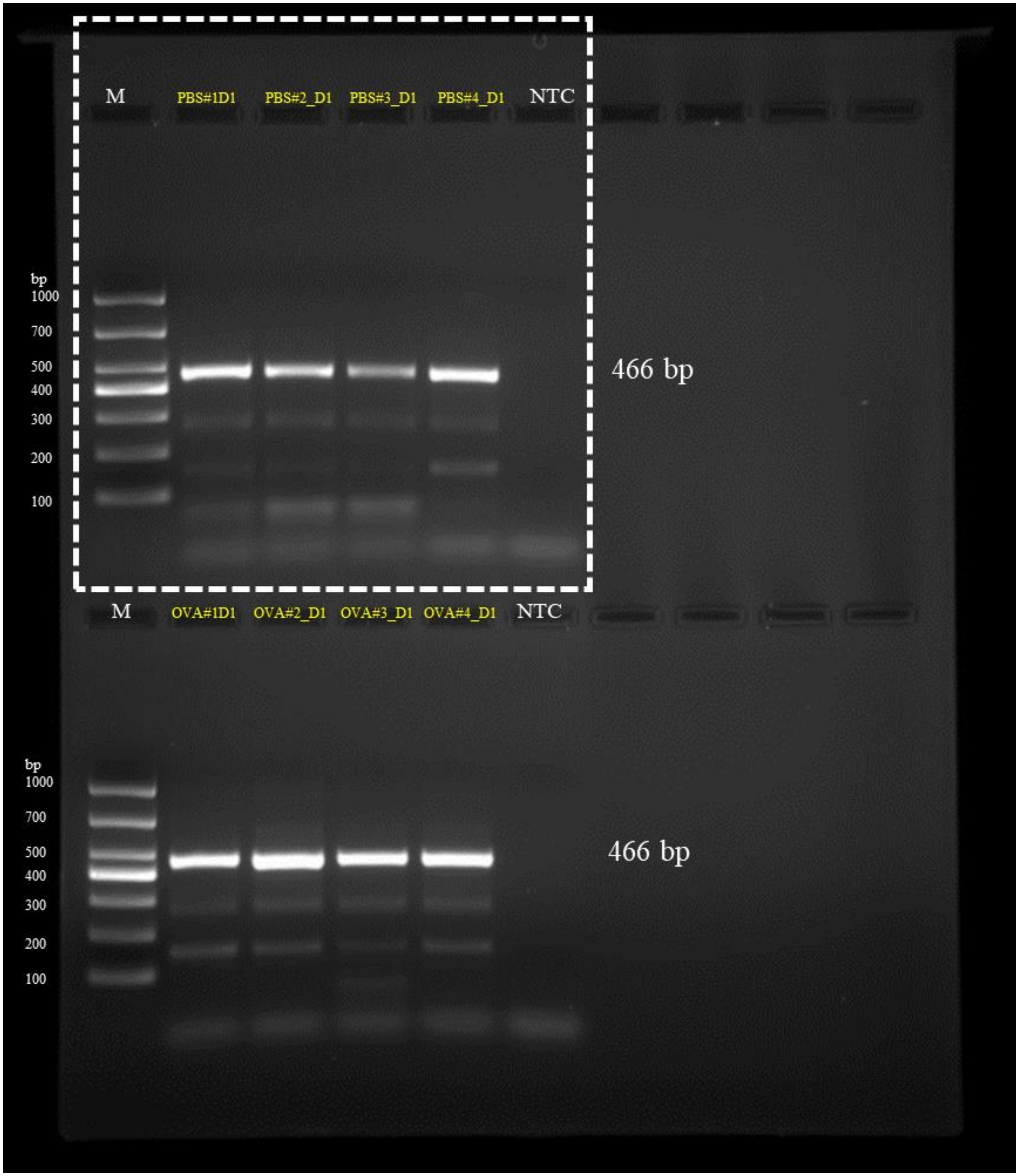

Supplementary Fig. S2. Full-length gel of Figure 3A showing the quality of 16S rRNA gene (V3-V4) extracted and amplified from the stool samples of the mice. The white dotted lines delineate the cropped region of the gel. M, DNA ladder DL1000; NTC, negative control; PBS, healthy control; OVA, OVA-induced allergic asthmatic control; D1, day 1.
